# (*E*)-*N*-[(5-Methyl-2-fur­yl)methyl­ene]-3-nitro­aniline

**DOI:** 10.1107/S1600536809007107

**Published:** 2009-03-06

**Authors:** Ya-Ning Guo

**Affiliations:** a Department of Chemistry, Baoji University of Arts and Sciences, Baoji, Shaanxi 721007, People’s Republic of China

## Abstract

The asymmetric unit of the title compound, C_12_H_10_N_2_O_3_, contains two crystallographically independent mol­ecules, in which the furan and benzene rings are oriented at dihedral angles of 46.09 (3) and 39.98 (3)°. In the crystal structure, weak inter­molecular C—H⋯N hydrogen bonds link the mol­ecules into chains running nearly parallel to the *a* axis.

## Related literature

For general background, see: Li & Zhang (2005 ▶); Antal *et al.* (1991 ▶); Basta & El-Saied (2003 ▶). For bond-length data, see: Allen *et al.* (1987 ▶).
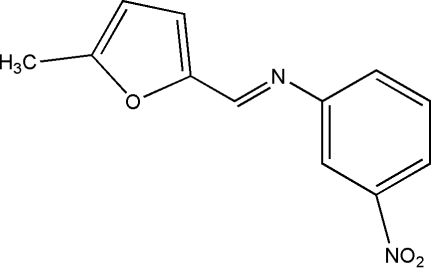


## Experimental

### 

#### Crystal data

C_12_H_10_N_2_O_3_
*M* *_r_* = 230.22
Orthorhombic, 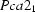

*a* = 21.634 (2) Å
*b* = 3.8286 (9) Å
*c* = 26.660 (2) Å
*V* = 2208.2 (6) Å^3^
*Z* = 8
Mo *K*α radiation
μ = 0.10 mm^−1^
*T* = 298 K
0.42 × 0.39 × 0.20 mm

#### Data collection

Bruker SMART CCD area-detector diffractometer
Absorption correction: multi-scan (*SADABS*; Siemens, 1996 ▶) *T*_min_ = 0.959, *T*_max_ = 0.980
8789 measured reflections
2002 independent reflections
1331 reflections with *I* > 2σ(*I*)
*R*_int_ = 0.051

#### Refinement

*R*[*F* ^2^ > 2σ(*F* ^2^)] = 0.045
*wR*(*F* ^2^) = 0.127
*S* = 1.06
2002 reflections
307 parameters
1 restraint
H-atom parameters constrained
Δρ_max_ = 0.15 e Å^−3^
Δρ_min_ = −0.16 e Å^−3^

### 

Data collection: *SMART* (Siemens, 1996 ▶); cell refinement: *SAINT* (Siemens, 1996 ▶); data reduction: *SAINT*; program(s) used to solve structure: *SHELXS97* (Sheldrick, 2008 ▶); program(s) used to refine structure: *SHELXL97* (Sheldrick, 2008 ▶); molecular graphics: *SHELXTL* (Sheldrick, 2008 ▶) and *ORTEP-3 for Windows* (Farrugia, 1997 ▶); software used to prepare material for publication: *SHELXTL*.

## Supplementary Material

Crystal structure: contains datablocks I, global. DOI: 10.1107/S1600536809007107/hk2629sup1.cif

Structure factors: contains datablocks I. DOI: 10.1107/S1600536809007107/hk2629Isup2.hkl

Additional supplementary materials:  crystallographic information; 3D view; checkCIF report

## Figures and Tables

**Table 1:** Hydrogen-bond geometry (Å, °)

*D*—H⋯*A*	*D*—H	H⋯*A*	*D*⋯*A*	*D*—H⋯*A*
C23—H23⋯N1^i^	0.93	2.51	3.429 (3)	172

Symmetry code: (i) 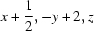.

**Fig. 1.**
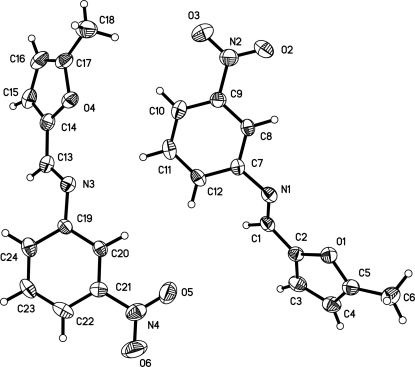
The molecular structure of the title molecule, with the atom-numbering scheme. Displacement ellipsoids are drawn at the 30% probability level.

**Fig. 2.**
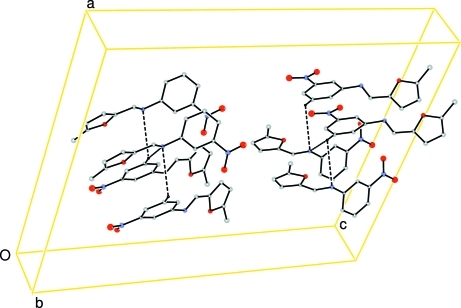
A partial packing diagram of the title compound. Hydrogen bonds are shown as dashed lines. H atoms not involved in hydrogen bonding are omitted.

## supplementary crystallographic information

### Comment

Schiff base complexes are of great interests for inorganic and bioinorganic chemists. To the best of our knowledge, in the past two decades, Schiff base complexes derived from furaldehydes have been less reported (Li & Zhang, 2005). Furaldehydes are raw materials used for preparing many medicines and industrial products, and some of furfural derivatives have a strong bactericidal ability and their antibacterial activities are fairly broad (Antal *et al.*, 1991; Basta & EI-Saied, 2003). As an extension of our work on the structural characterizations of Schiff bases of furaldehyde derivatives, we report herein the crystal structure of the title compound.

The asymmetric unit of the title compound contains two crystallographically independent molecules (Fig. 1), in which the bond lengths (Allen *et al.*, 1987) and angles are generally within normal ranges. Rings A (O1/C2-C5), B (C7-C12) and C (O4/C14-C17), D (C19-C24) are, of course, planar, and they are oriented at dihedral angles of A/B = 46.09 (3) and C/D = 39.98 (3) °.

In the crystal structure, weak intermolecular C-H···N hydrogen bonds (Table 1) link the molecules into chains nearly parallel to the a-axis (Fig. 2), in which they may be effective in the stabilization of the structure.

### Experimental

For the preparation of the title compound, 5-methyl-2-furaldehyde (11.0 mg, 0.1 mmol) and 3-nitrobenzenamine (13.8 mg, 0.1 mmol) were dissolved in methanol (10 ml). The mixture was stirred for 1 h at room temperature, and then filtered. After allowing the filtrate to stand in air for 7 d, yellow block-shaped crystals of the title compound were obtained. They were collected, washed with methanol and dried in a vacuum desiccator using anhydrous CaCl_2_ (yield; 60%).

### Refinement

H atoms were positioned geometrically, with C-H = 0.93 and 0.96 Å for aromatic and methyl H, respectively, and constrained to ride on their parent atoms, with U_iso_(H) = xU_eq_(C), where x = 1.2 for aromatic H and x = 1.5 for methyl H atoms. Friedel pairs were merged.

### Crystal data

**Table:**

C_12_H_10_N_2_O_3_	*F*(000) = 960
*M**_r_* = 230.22	*D*_x_ = 1.385 Mg m^−^^3^
Orthorhombic, *P**c**a*2_1_	Mo *K*α radiation, λ = 0.71073 Å
Hall symbol: P 2c -2ac	Cell parameters from 1515 reflections
*a* = 21.634 (2) Å	θ = 2.4–20.5°
*b* = 3.8286 (9) Å	µ = 0.10 mm^−^^1^
*c* = 26.660 (2) Å	*T* = 298 K
*V* = 2208.2 (6) Å^3^	Block, yellow
*Z* = 8	0.42 × 0.39 × 0.20 mm

### Data collection

**Table:**

Bruker SMART CCD area-detector diffractometer	2002 independent reflections
Radiation source: fine-focus sealed tube	1331 reflections with *I* > 2σ(*I*)
graphite	*R*_int_ = 0.051
φ and ω scans	θ_max_ = 25.0°, θ_min_ = 1.5°
Absorption correction: multi-scan (*SADABS*; Siemens, 1996)	*h* = −25→24
*T*_min_ = 0.959, *T*_max_ = 0.980	*k* = −4→4
8789 measured reflections	*l* = −31→23

### Refinement

**Table:**

Refinement on *F*^2^	Primary atom site location: structure-invariant direct methods
Least-squares matrix: full	Secondary atom site location: difference Fourier map
*R*[*F*^2^ > 2σ(*F*^2^)] = 0.045	Hydrogen site location: inferred from neighbouring sites
*wR*(*F*^2^) = 0.127	H-atom parameters constrained
*S* = 1.06	*w* = 1/[σ^2^(*F*_o_^2^) + (0.0216*P*)^2^ + 0.7218*P*] where *P* = (*F*_o_^2^ + 2*F*_c_^2^)/3
2002 reflections	(Δ/σ)_max_ < 0.001
307 parameters	Δρ_max_ = 0.15 e Å^−^^3^
1 restraint	Δρ_min_ = −0.15 e Å^−^^3^

### Special details

**Table:**

Geometry. All e.s.d.'s (except the e.s.d. in the dihedral angle between two l.s. planes) are estimated using the full covariance matrix. The cell e.s.d.'s are taken into account individually in the estimation of e.s.d.'s in distances, angles and torsion angles; correlations between e.s.d.'s in cell parameters are only used when they are defined by crystal symmetry. An approximate (isotropic) treatment of cell e.s.d.'s is used for estimating e.s.d.'s involving l.s. planes.
Refinement. Refinement of *F*^2^ against ALL reflections. The weighted *R*-factor *wR* and goodness of fit *S* are based on *F*^2^, conventional *R*-factors *R* are based on *F*, with *F* set to zero for negative *F*^2^. The threshold expression of *F*^2^ > σ(*F*^2^) is used only for calculating *R*-factors(gt) *etc*. and is not relevant to the choice of reflections for refinement. *R*-factors based on *F*^2^ are statistically about twice as large as those based on *F*, and *R*- factors based on ALL data will be even larger.

### Fractional atomic coordinates and isotropic or equivalent isotropic displacement parameters (Å^2^)

**Table:**

	*x*	*y*	*z*	*U*_iso_*/*U*_eq_	
N1	0.06199 (19)	0.3004 (10)	0.26104 (17)	0.0472 (11)	
N2	0.0529 (3)	0.1073 (14)	0.4392 (2)	0.0681 (14)	
N3	0.31807 (19)	0.9617 (11)	0.36859 (16)	0.0470 (11)	
N4	0.2860 (3)	1.0358 (15)	0.19047 (19)	0.0657 (14)	
O1	0.00925 (16)	0.2744 (9)	0.16401 (13)	0.0497 (9)	
O2	0.0034 (2)	−0.0350 (15)	0.43257 (19)	0.0921 (16)	
O3	0.0753 (3)	0.1515 (18)	0.48052 (16)	0.110 (2)	
O4	0.27482 (18)	0.7909 (9)	0.46480 (13)	0.0520 (10)	
O5	0.2380 (2)	0.8701 (14)	0.19661 (19)	0.0890 (16)	
O6	0.3026 (3)	1.1466 (17)	0.14984 (17)	0.1053 (19)	
C1	0.0915 (2)	0.1827 (14)	0.2234 (2)	0.0466 (13)	
H1	0.1320	0.1096	0.2287	0.056*	
C2	0.0677 (2)	0.1540 (14)	0.17390 (19)	0.0460 (13)	
C3	0.0929 (3)	0.0267 (15)	0.1313 (2)	0.0586 (16)	
H3	0.1321	−0.0699	0.1277	0.070*	
C4	0.0488 (3)	0.0683 (15)	0.0937 (2)	0.0611 (16)	
H4	0.0533	0.0052	0.0602	0.073*	
C5	−0.0012 (3)	0.2153 (14)	0.1144 (2)	0.0526 (15)	
C6	−0.0618 (3)	0.3237 (16)	0.0943 (2)	0.0646 (17)	
H6A	−0.0631	0.2783	0.0589	0.097*	
H6B	−0.0677	0.5688	0.1001	0.097*	
H6C	−0.0940	0.1944	0.1107	0.097*	
C7	0.0929 (2)	0.3138 (12)	0.3078 (2)	0.0409 (12)	
C8	0.0596 (2)	0.2124 (14)	0.3490 (2)	0.0447 (13)	
H8	0.0189	0.1385	0.3459	0.054*	
C9	0.0879 (3)	0.2223 (15)	0.39510 (19)	0.0494 (14)	
C10	0.1472 (3)	0.3357 (16)	0.4017 (2)	0.0592 (15)	
H10	0.1653	0.3391	0.4334	0.071*	
C11	0.1794 (3)	0.4448 (15)	0.3600 (2)	0.0609 (16)	
H11	0.2196	0.5280	0.3635	0.073*	
C12	0.1525 (2)	0.4314 (13)	0.3136 (2)	0.0499 (14)	
H12	0.1749	0.5026	0.2856	0.060*	
C13	0.3537 (3)	0.8338 (15)	0.4012 (2)	0.0511 (14)	
H13	0.3951	0.8058	0.3929	0.061*	
C14	0.3343 (3)	0.7292 (14)	0.4502 (2)	0.0490 (13)	
C15	0.3657 (3)	0.5765 (16)	0.4878 (2)	0.0621 (16)	
H15	0.4070	0.5101	0.4873	0.075*	
C16	0.3243 (3)	0.5366 (16)	0.5277 (2)	0.0672 (18)	
H16	0.3329	0.4364	0.5587	0.081*	
C17	0.2701 (3)	0.6692 (14)	0.5130 (2)	0.0569 (16)	
C18	0.2091 (3)	0.7113 (18)	0.5369 (2)	0.078 (2)	
H18A	0.2017	0.9543	0.5434	0.117*	
H18B	0.2082	0.5841	0.5679	0.117*	
H18C	0.1776	0.6229	0.5149	0.117*	
C19	0.3425 (2)	1.0521 (13)	0.32122 (18)	0.0421 (12)	
C20	0.3048 (2)	1.0067 (12)	0.27975 (19)	0.0422 (12)	
H20	0.2651	0.9172	0.2836	0.051*	
C21	0.3259 (3)	1.0933 (14)	0.23347 (19)	0.0484 (13)	
C22	0.3846 (3)	1.2301 (14)	0.2259 (2)	0.0589 (16)	
H22	0.3984	1.2841	0.1937	0.071*	
C23	0.4218 (3)	1.2838 (15)	0.2669 (3)	0.0568 (15)	
H23	0.4611	1.3780	0.2629	0.068*	
C24	0.4007 (2)	1.1980 (13)	0.3141 (2)	0.0542 (14)	
H24	0.4259	1.2383	0.3417	0.065*	

### Atomic displacement parameters (Å^2^)

**Table:**

	*U*^11^	*U*^22^	*U*^33^	*U*^12^	*U*^13^	*U*^23^
N1	0.040 (2)	0.046 (3)	0.055 (3)	0.002 (2)	0.007 (2)	0.003 (2)
N2	0.060 (4)	0.085 (4)	0.059 (4)	0.005 (3)	0.004 (3)	0.008 (3)
N3	0.047 (3)	0.047 (3)	0.047 (3)	0.000 (2)	−0.002 (2)	−0.003 (2)
N4	0.065 (4)	0.083 (4)	0.048 (3)	0.019 (3)	0.004 (3)	−0.002 (3)
O1	0.050 (2)	0.050 (2)	0.049 (2)	0.0024 (17)	0.0033 (17)	−0.0044 (17)
O2	0.060 (3)	0.141 (5)	0.076 (3)	−0.015 (3)	0.015 (2)	0.016 (3)
O3	0.116 (4)	0.164 (6)	0.052 (3)	−0.029 (4)	−0.012 (3)	0.018 (3)
O4	0.059 (3)	0.053 (2)	0.044 (2)	0.0019 (18)	−0.0083 (17)	0.0021 (18)
O5	0.063 (3)	0.132 (5)	0.072 (3)	−0.004 (3)	−0.013 (2)	−0.014 (3)
O6	0.128 (5)	0.140 (5)	0.048 (3)	0.002 (4)	0.006 (3)	0.007 (3)
C1	0.033 (3)	0.050 (3)	0.057 (4)	−0.001 (2)	0.006 (3)	0.006 (3)
C2	0.044 (3)	0.048 (3)	0.047 (3)	0.003 (2)	0.006 (3)	0.001 (2)
C3	0.058 (4)	0.055 (4)	0.062 (4)	0.003 (3)	0.014 (3)	−0.002 (3)
C4	0.075 (4)	0.058 (4)	0.050 (3)	−0.007 (3)	0.014 (3)	−0.010 (3)
C5	0.063 (4)	0.048 (3)	0.047 (4)	−0.009 (3)	0.003 (3)	−0.004 (3)
C6	0.076 (5)	0.062 (4)	0.055 (4)	0.000 (3)	−0.007 (3)	−0.002 (3)
C7	0.036 (3)	0.035 (3)	0.051 (3)	0.004 (2)	−0.002 (3)	−0.003 (2)
C8	0.034 (3)	0.045 (3)	0.055 (3)	0.002 (2)	−0.001 (3)	−0.002 (3)
C9	0.049 (3)	0.052 (4)	0.046 (3)	0.002 (3)	0.002 (3)	0.002 (3)
C10	0.054 (4)	0.064 (4)	0.060 (4)	0.002 (3)	−0.016 (3)	−0.005 (3)
C11	0.040 (3)	0.061 (4)	0.082 (5)	−0.010 (3)	−0.009 (3)	−0.002 (3)
C12	0.041 (3)	0.042 (3)	0.067 (4)	−0.006 (2)	0.005 (3)	0.007 (3)
C13	0.051 (4)	0.050 (3)	0.052 (4)	−0.002 (3)	−0.007 (3)	−0.007 (3)
C14	0.051 (3)	0.043 (3)	0.052 (3)	0.003 (3)	−0.007 (3)	−0.008 (3)
C15	0.066 (4)	0.053 (4)	0.067 (4)	−0.001 (3)	−0.025 (3)	−0.004 (3)
C16	0.097 (5)	0.060 (4)	0.045 (4)	−0.002 (4)	−0.020 (4)	0.002 (3)
C17	0.087 (5)	0.044 (3)	0.040 (3)	−0.009 (3)	−0.003 (3)	−0.003 (3)
C18	0.097 (5)	0.072 (5)	0.064 (4)	−0.011 (4)	0.016 (4)	0.000 (3)
C19	0.041 (3)	0.039 (3)	0.046 (3)	−0.004 (2)	0.007 (2)	−0.001 (2)
C20	0.033 (3)	0.043 (3)	0.051 (3)	0.000 (2)	0.005 (2)	−0.002 (3)
C21	0.053 (3)	0.044 (3)	0.048 (3)	0.007 (3)	0.007 (3)	0.002 (3)
C22	0.062 (4)	0.044 (3)	0.071 (4)	0.002 (3)	0.027 (4)	0.010 (3)
C23	0.039 (3)	0.049 (4)	0.083 (4)	−0.008 (2)	0.014 (3)	0.000 (3)
C24	0.046 (3)	0.044 (3)	0.073 (4)	0.000 (2)	0.002 (3)	−0.010 (3)

### Geometric parameters (Å, °)

**Table:**

N1—C1	1.273 (6)	C8—H8	0.9300
N1—C7	1.415 (6)	C9—C10	1.366 (7)
N2—O2	1.215 (7)	C10—C11	1.376 (8)
N2—O3	1.216 (6)	C10—H10	0.9300
N2—C9	1.465 (7)	C11—C12	1.369 (8)
N3—C13	1.260 (6)	C11—H11	0.9300
N3—C19	1.412 (6)	C12—H12	0.9300
N4—O6	1.218 (7)	C13—C14	1.429 (8)
N4—O5	1.229 (6)	C13—H13	0.9300
N4—C21	1.452 (7)	C14—C15	1.345 (8)
O1—C5	1.359 (6)	C15—C16	1.400 (9)
O1—C2	1.371 (6)	C15—H15	0.9300
O4—C14	1.364 (7)	C16—C17	1.335 (8)
O4—C17	1.372 (6)	C16—H16	0.9300
C1—C2	1.420 (7)	C17—C18	1.474 (9)
C1—H1	0.9300	C18—H18A	0.9600
C2—C3	1.352 (7)	C18—H18B	0.9600
C3—C4	1.393 (9)	C18—H18C	0.9600
C3—H3	0.9300	C19—C20	1.385 (7)
C4—C5	1.339 (8)	C19—C24	1.391 (7)
C4—H4	0.9300	C20—C21	1.357 (7)
C5—C6	1.477 (8)	C20—H20	0.9300
C6—H6A	0.9600	C21—C22	1.387 (8)
C6—H6B	0.9600	C22—C23	1.375 (8)
C6—H6C	0.9600	C22—H22	0.9300
C7—C8	1.370 (7)	C23—C24	1.377 (8)
C7—C12	1.375 (6)	C23—H23	0.9300
C8—C9	1.372 (7)	C24—H24	0.9300
			
C1—N1—C7	118.1 (4)	C12—C11—H11	119.9
O2—N2—O3	123.1 (6)	C10—C11—H11	119.9
O2—N2—C9	118.3 (5)	C11—C12—C7	120.9 (5)
O3—N2—C9	118.7 (6)	C11—C12—H12	119.6
C13—N3—C19	118.9 (5)	C7—C12—H12	119.6
O6—N4—O5	123.2 (6)	N3—C13—C14	124.0 (5)
O6—N4—C21	118.3 (6)	N3—C13—H13	118.0
O5—N4—C21	118.4 (5)	C14—C13—H13	118.0
C5—O1—C2	106.5 (4)	C15—C14—O4	109.8 (5)
C14—O4—C17	106.2 (4)	C15—C14—C13	130.9 (6)
N1—C1—C2	125.3 (5)	O4—C14—C13	119.3 (5)
N1—C1—H1	117.4	C14—C15—C16	106.9 (6)
C2—C1—H1	117.4	C14—C15—H15	126.6
C3—C2—O1	109.4 (5)	C16—C15—H15	126.6
C3—C2—C1	131.5 (5)	C17—C16—C15	107.3 (5)
O1—C2—C1	119.2 (4)	C17—C16—H16	126.3
C2—C3—C4	106.7 (5)	C15—C16—H16	126.3
C2—C3—H3	126.6	C16—C17—O4	109.8 (5)
C4—C3—H3	126.6	C16—C17—C18	134.5 (6)
C5—C4—C3	107.7 (5)	O4—C17—C18	115.7 (6)
C5—C4—H4	126.2	C17—C18—H18A	109.5
C3—C4—H4	126.2	C17—C18—H18B	109.5
C4—C5—O1	109.7 (5)	H18A—C18—H18B	109.5
C4—C5—C6	133.2 (6)	C17—C18—H18C	109.5
O1—C5—C6	117.1 (5)	H18A—C18—H18C	109.5
C5—C6—H6A	109.5	H18B—C18—H18C	109.5
C5—C6—H6B	109.5	C20—C19—C24	118.3 (5)
H6A—C6—H6B	109.5	C20—C19—N3	117.6 (4)
C5—C6—H6C	109.5	C24—C19—N3	124.0 (5)
H6A—C6—H6C	109.5	C21—C20—C19	119.8 (5)
H6B—C6—H6C	109.5	C21—C20—H20	120.1
C8—C7—C12	119.6 (5)	C19—C20—H20	120.1
C8—C7—N1	116.7 (4)	C20—C21—C22	122.2 (5)
C12—C7—N1	123.7 (5)	C20—C21—N4	118.7 (5)
C7—C8—C9	118.5 (5)	C22—C21—N4	119.1 (5)
C7—C8—H8	120.8	C23—C22—C21	118.4 (5)
C9—C8—H8	120.8	C23—C22—H22	120.8
C10—C9—C8	122.8 (5)	C21—C22—H22	120.8
C10—C9—N2	118.5 (5)	C22—C23—C24	119.9 (5)
C8—C9—N2	118.7 (5)	C22—C23—H23	120.1
C9—C10—C11	117.9 (5)	C24—C23—H23	120.1
C9—C10—H10	121.0	C23—C24—C19	121.4 (5)
C11—C10—H10	121.0	C23—C24—H24	119.3
C12—C11—C10	120.2 (5)	C19—C24—H24	119.3
			
C7—N1—C1—C2	−179.5 (5)	C19—N3—C13—C14	−179.4 (5)
C5—O1—C2—C3	0.6 (6)	C17—O4—C14—C15	−0.3 (6)
C5—O1—C2—C1	179.8 (5)	C17—O4—C14—C13	−179.1 (5)
N1—C1—C2—C3	−177.9 (6)	N3—C13—C14—C15	176.7 (6)
N1—C1—C2—O1	3.1 (8)	N3—C13—C14—O4	−4.8 (8)
O1—C2—C3—C4	−0.1 (6)	O4—C14—C15—C16	0.6 (7)
C1—C2—C3—C4	−179.2 (6)	C13—C14—C15—C16	179.2 (5)
C2—C3—C4—C5	−0.4 (7)	C14—C15—C16—C17	−0.7 (7)
C3—C4—C5—O1	0.7 (6)	C15—C16—C17—O4	0.5 (7)
C3—C4—C5—C6	179.7 (6)	C15—C16—C17—C18	−179.2 (6)
C2—O1—C5—C4	−0.8 (6)	C14—O4—C17—C16	−0.2 (6)
C2—O1—C5—C6	−180.0 (5)	C14—O4—C17—C18	179.6 (5)
C1—N1—C7—C8	−137.9 (5)	C13—N3—C19—C20	145.3 (5)
C1—N1—C7—C12	44.1 (7)	C13—N3—C19—C24	−37.8 (7)
C12—C7—C8—C9	−1.8 (7)	C24—C19—C20—C21	2.2 (7)
N1—C7—C8—C9	−179.9 (4)	N3—C19—C20—C21	179.3 (5)
C7—C8—C9—C10	1.4 (8)	C19—C20—C21—C22	−0.5 (8)
C7—C8—C9—N2	−178.8 (5)	C19—C20—C21—N4	178.6 (5)
O2—N2—C9—C10	−170.6 (6)	O6—N4—C21—C20	172.6 (6)
O3—N2—C9—C10	7.8 (9)	O5—N4—C21—C20	−9.2 (8)
O2—N2—C9—C8	9.5 (8)	O6—N4—C21—C22	−8.3 (8)
O3—N2—C9—C8	−172.1 (6)	O5—N4—C21—C22	169.9 (5)
C8—C9—C10—C11	0.2 (9)	C20—C21—C22—C23	−1.1 (8)
N2—C9—C10—C11	−179.7 (5)	N4—C21—C22—C23	179.9 (5)
C9—C10—C11—C12	−1.3 (9)	C21—C22—C23—C24	0.8 (8)
C10—C11—C12—C7	0.9 (8)	C22—C23—C24—C19	0.9 (8)
C8—C7—C12—C11	0.7 (8)	C20—C19—C24—C23	−2.4 (8)
N1—C7—C12—C11	178.6 (5)	N3—C19—C24—C23	−179.4 (5)

### Hydrogen-bond geometry (Å, °)

**Table:**

*D*—H···*A*	*D*—H	H···*A*	*D*···*A*	*D*—H···*A*
C23—H23···N1^i^	0.93	2.51	3.429 (3)	172

Symmetry codes: (i) *x*+1/2, −*y*+2, *z*.
